# Supplementary material for: Single-molecule epitranscriptomic analysis of full-length HIV-1 RNAs reveals functional roles of site-specific m6As
Source: Nat Microbiol. 2024 Apr 11;9(5):1340–55. doi: 10.1038/s41564-024-01638-5 (PMC11087264; doi:10.1038/s41564-024-01638-5)
Supplement: Supplementary file 1 — Supplementary Figs. 1–8. [file 41564_2024_1638_MOESM1_ESM.pdf]

nature microbiology

Article

https://doi.org/10.1038/s41564-024-01638-5

Single-molecule epitranscriptomic analysis of full-length HIV-1 RNAs reveals functional roles of site-specific m^{6}As

In the format provided by the authors and unedited

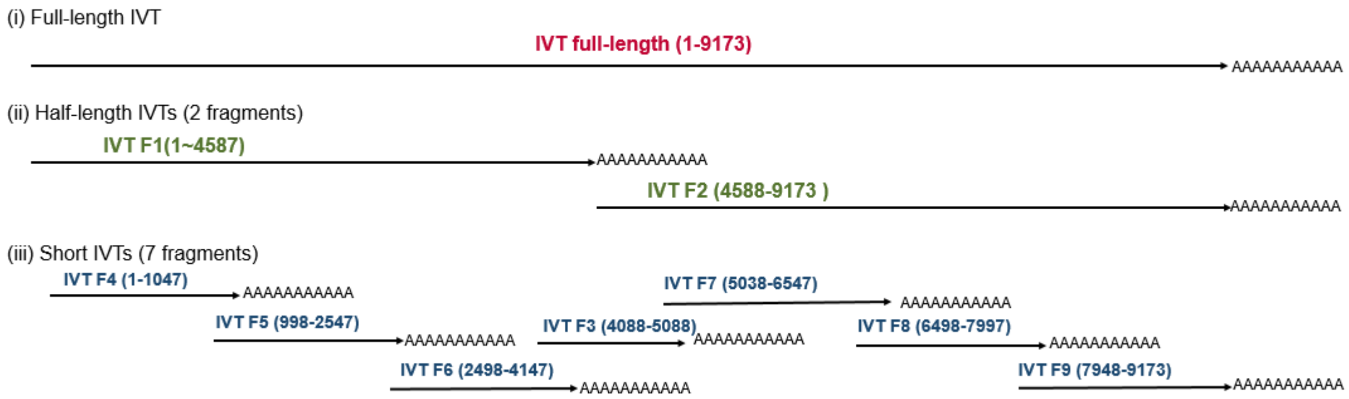

Supplementary Fig. 1. Schematic view of IVT RNA controls. DNA templates were generated with the Sp6 promotor at the 5' end by PCR amplification using the primer sets in **Supplementary Table 1.** The PCR amplicons were then gel-purified (QIAquick gel extraction kit, Qiagen), and subjected for in vitro RNA transcription (RiboMAX™ Large Scale RNA Production Systems, Promega). After purification (RNA Clean XP, Beckman Coulter), 1ug of IVT RNAs were subjected to ONT DRS using ONT DRS kit (SQK-RNA002).

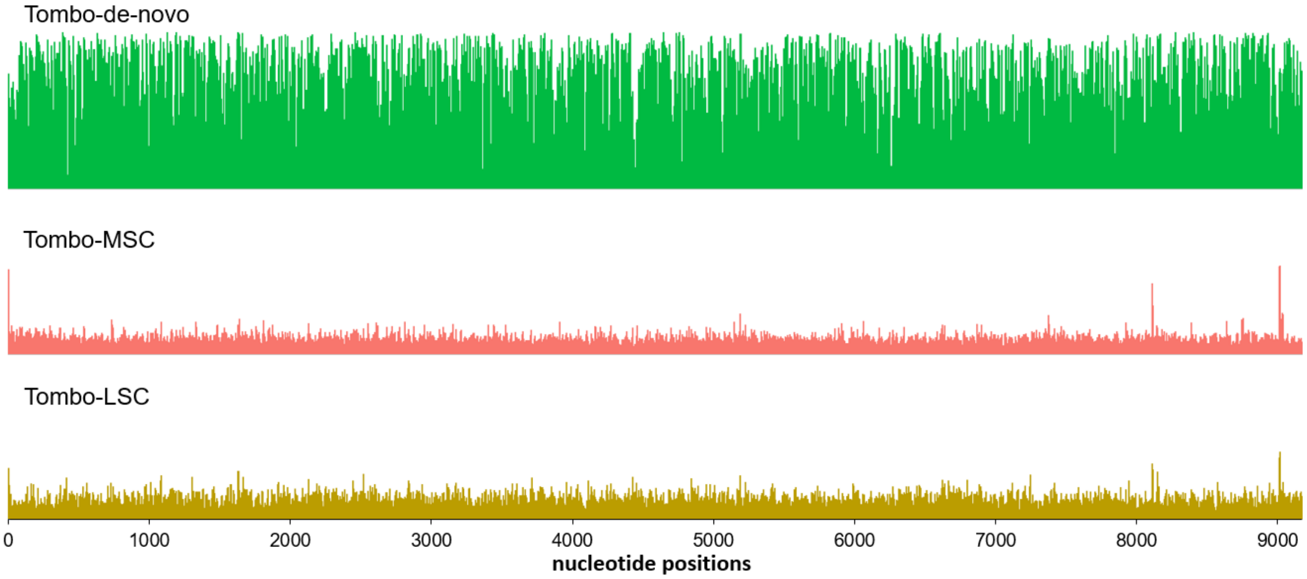

Supplementary Fig. 2. Noise-reduction using Tombo-MSC (Step 1). 3,985 reads of full-length virion RNA were analyzed with Tombo de-novo (top panel), model-sample-compare (MSC; middle panel), and level-sample-compare (LSC; bottom panel) options following the instructions (https://github.com/nanoporetech/tombo)[1]. 5,411 reads of full-length (> 8 Kb) IVT RNA were used as a canonical control for Tombo-MSC and for Tombo-level-sample-compare (Tombo-LSC).

(i)

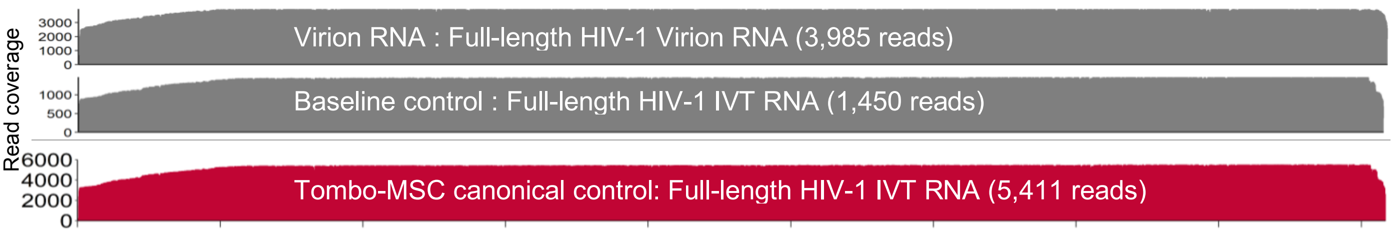

(ii)

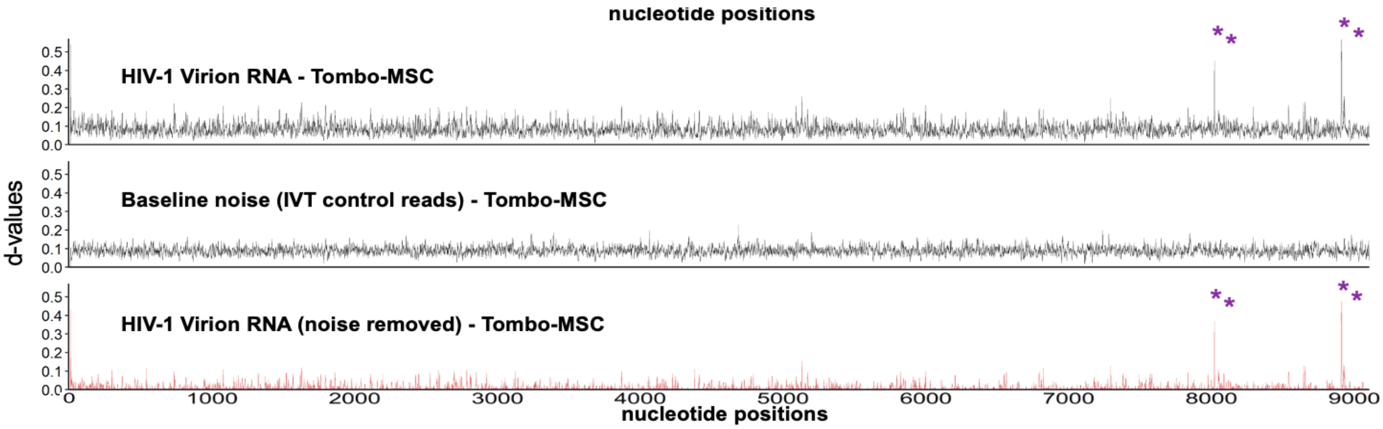

Supplementary Fig. 3. Removal of the baseline noise using IVT subreads (Step 2). (i) A total of 3,985 virion RNA reads and 1,450 IVT RNA reads were analyzed with Tombo-MSC using 5,411 reads of canonical control. The baseline control reads (1,450 IVT subreads) were not used as a canonical control. (ii) Tombo-MSC data (d-values, y-axis) for HIV-1 virion RNA (top panel) and full-length IVT subreads (middle panel, representing the baseline noise) were compared. A subtraction of the baseline noise substantially refined the d-values of HIV-1 virion RNA, identifying the four prominent modification peaks near the 3' end (purple asterisks on the bottom panel).

(**a**)

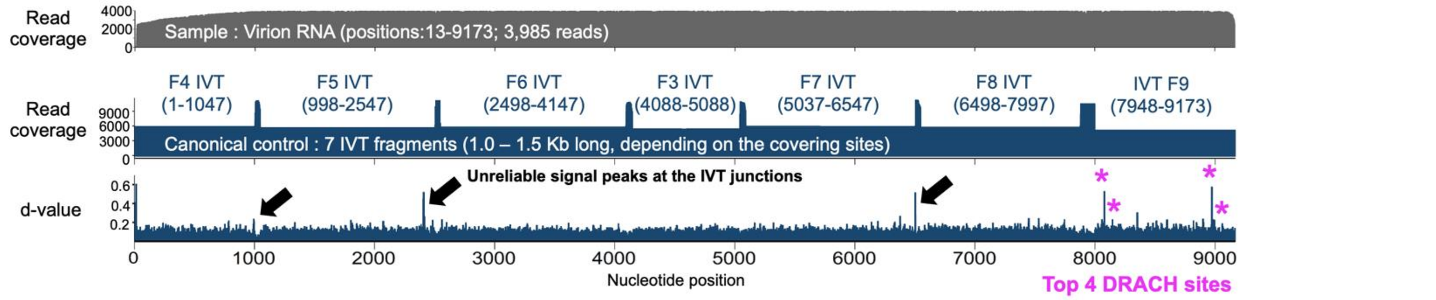

(**b**)

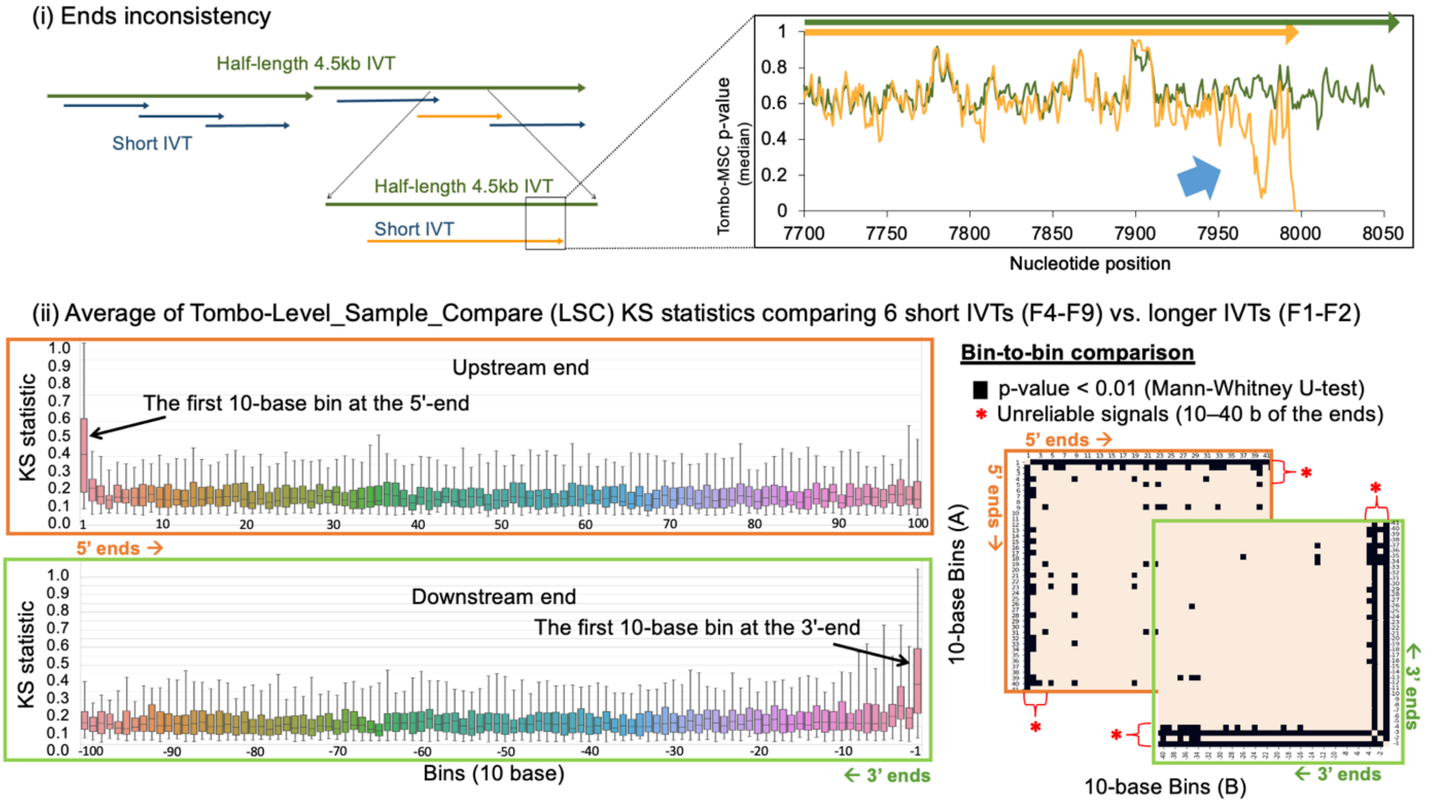

Supplementary Fig. 4. DRS signals near the end of each read are not reliable. (**a**) Short IVT controls generate unreliable DRS signals near the ends of RNA reads. Tombo-MSC analysis was performed for full-length virion RNA reads (grey; total 3,985 reads of >8 Kb) using IVT canonical control reads consisting of 7 fragments of 1-1.5 Kb IVT RNAs (F3-F9; approximately 6,000 reads each; see Supplementary Information Fig. 1-iii-). Strong d-value signals were found near the junctions of neighboring IVT canonical controls (black arrows), which were not seen in similar analysis with long IVT reads (Extended Data Fig. 2a). These peaks may be the results of the DRS signal instability at the 5' and 3' ends of each read. (**b**) DRS signal instability at the 5' and 3' ends. (i) Schematic view of the analysis to determine the DRS signal instability at the 5' and 3' ends of each read. We compared DRS signals (e.g. per-read p-values and current levels) of identical sequences between long IVT RNAs (F1 and F2) and short IVT RNAs (F4 – F9) to evaluate the quality of DRS signals at different positions. The right panel shows an example comparing F8 (orange) and F2 RNA (dark green). Signal deviations are evident near the 3' end of short IVT RNAs (light blue arrow). (ii) The stability of DRS signals was systemically evaluated at both ends. The KS-statistics for each position (y-axis; calculated by Tombo-"Level Sample Compare" or Tombo-LSC) were used as a measure of signal differences between the long and short IVT datasets. The box-plots on the left show KS-statistics values (aggregated over bins of 10 base) for 6 different

sets (F4 – F9 against F1-F2), plotted from the 5' ends (upper panel; orange box) and from the 3' ends (lower panel; green box). The right panel show Mann-Whitney U-tests comparing every pair of 10-base bins (black squares denote $p < 0.01$). We concluded the first 10-40 bases from either end (denoted by red asterisks) were generally unreliable, often showing significant differences ($p < 0.01$).

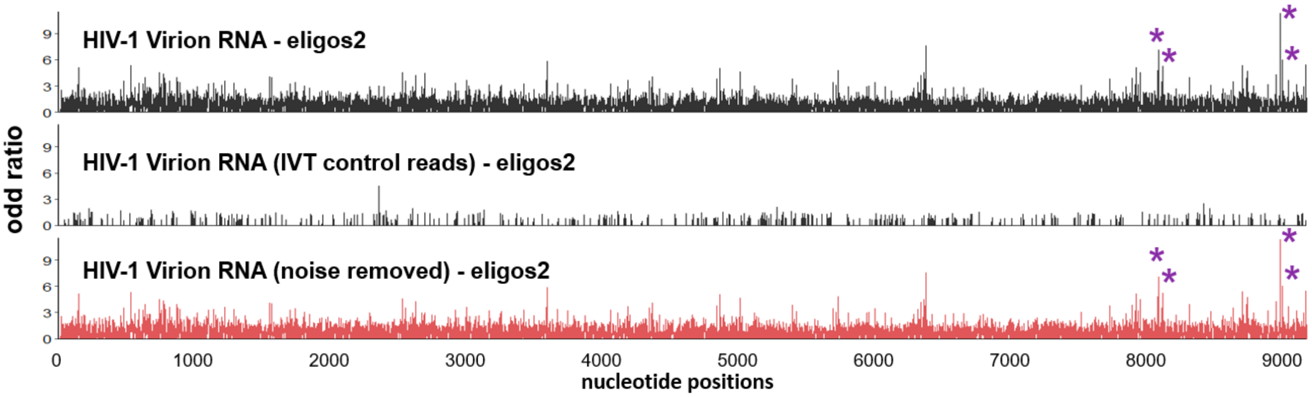

Supplementary Fig. 5. Eligos2 analysis of HIV-1 RNA modifications. Odd ratios (y-axis) from Eligos2 analysis for HIV-1 virion RNA (top panel) and full-length IVT subreads (middle panel, representing the baseline noise) were compared. A subtraction of the baseline noise substantially refined the modification signals of HIV-1 virion RNA, identifying the four prominent modification peaks near the 3' end (purple asterisks on the bottom panel).

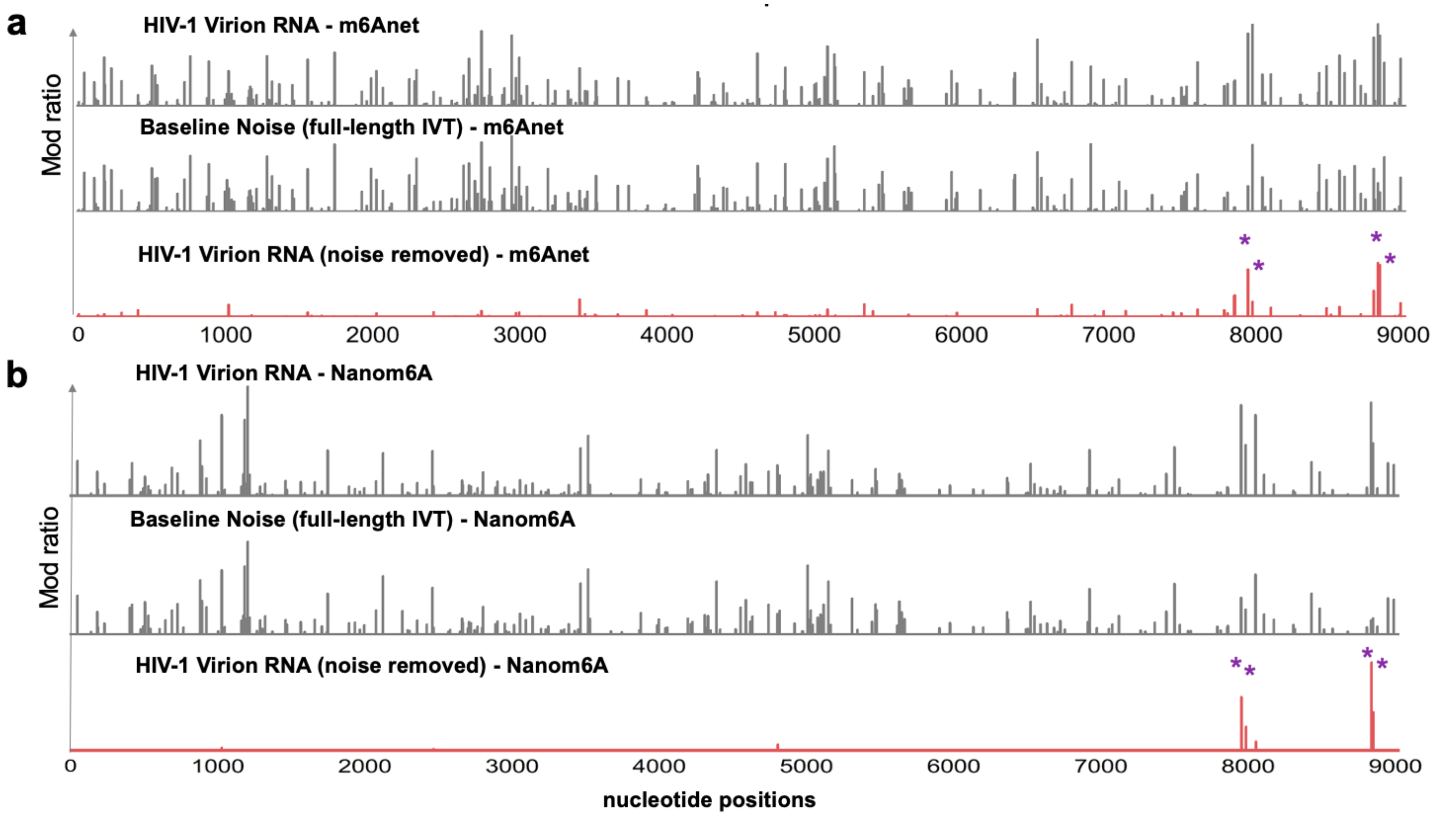

Supplementary Fig. 6. Nanom6A and m6Anet analyses of HIV-1 RNA m6As. (a-b) Modification ratios (Mod ratios, y-axis) estimated by m6Anet (**a**) and by nanom6A (**b**) are shown for HIV-1 virion RNA (top panel) and full-length IVT subreads (middle panel, representing the baseline noise). A subtraction of the baseline noise substantially refined the modification signals of HIV-1 virion RNA, identifying the four prominent m6As near the 3' end (purple asterisks on the bottom panel).

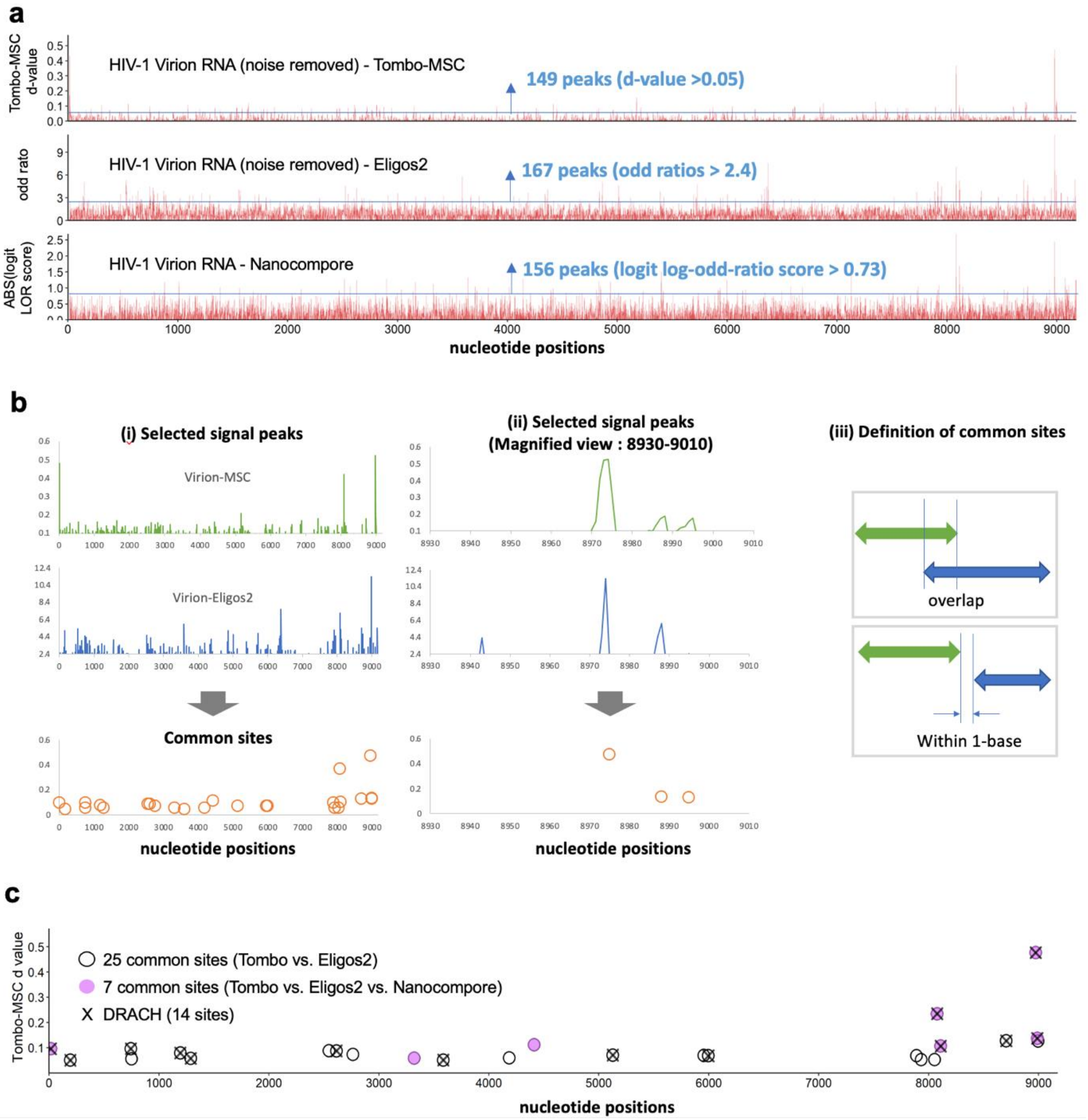

Supplementary Figure 7. Common modification sites among Tombo, Eligos2 and Nanocompore results. (**a**) Top 149, 167, and 156 most probable modification sites were chosen from Tombo-MSC (top panel), Eligos2 (middle panel), and Nanocompore data (bottom panel), respectively. (**b**) the selected modification peaks were cross-compared to determine the most probably modification sites. The common sites were determined when two peaks were either overlap or within 1 base distance (iii). (**c**) 25 common peaks between Tombo-MSC and Eligos2 (circles), and 7 common peaks among all three datasets (purple circles) are shown. 14 out of 25 common sites (From Tombo and Eligos2) are on DRACH sites (crosses). 5 out of 7 common sites (from Tombo, Eligos2 and Nanocompore) are on DRACH sites.

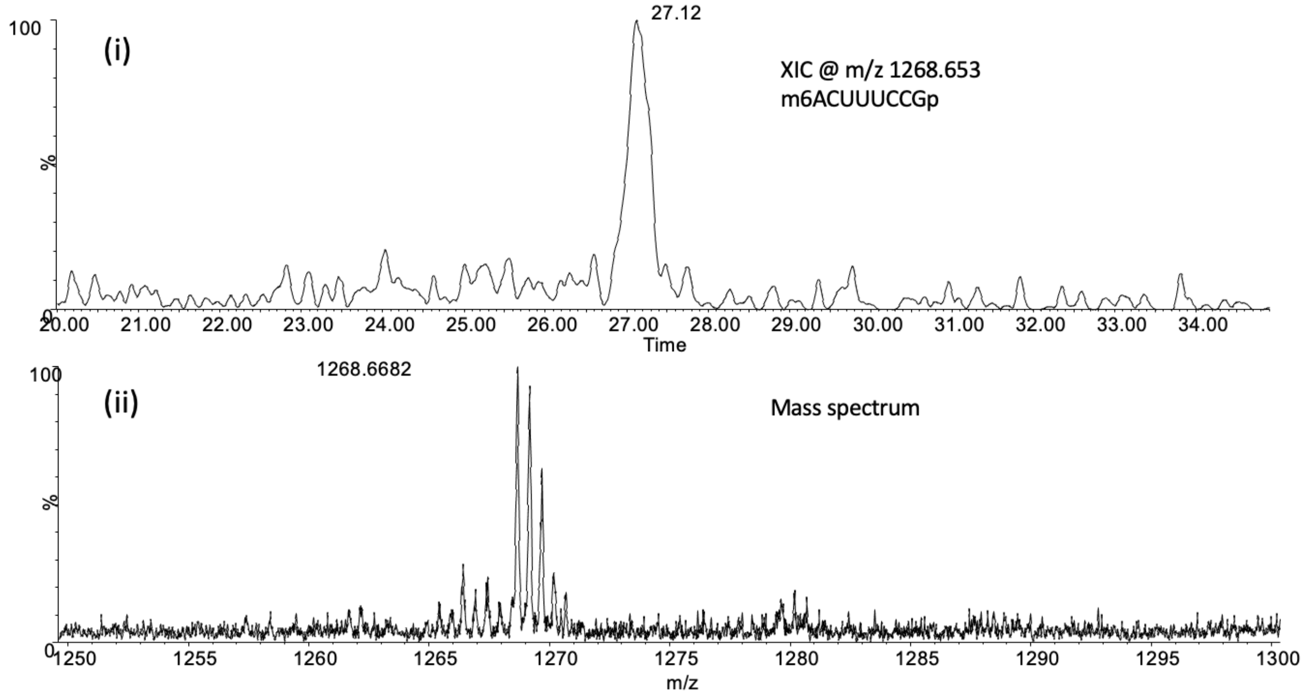

(b) m^{6}A8989 (m^{6}ACUUUCCAGp)

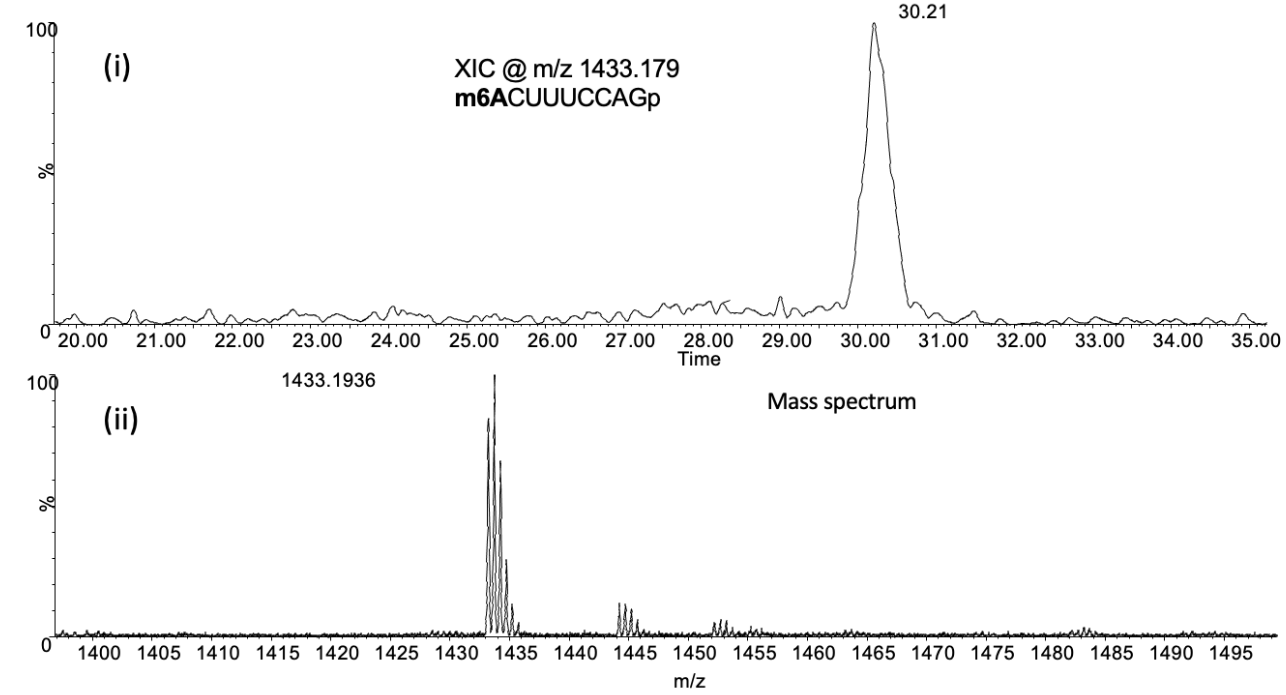

Supplementary Fig. 8. Oligonucleotide LC-MS/MS confirmed m^{6}A at 8989A and 8975A in HIV-1 viral RNA. Extracted ion chromatograms for methylated HIV-1 RNA oligomers corresponding to positions at 8975 (a-i-) and 8989 (b-i-). Mass spectra of the methylated oligonucleotides confirms m^{6}A at 8975A (a-ii-) and 8989A (b-ii-) in HIV-1 viral RNA. Sample preparation and data analysis were performed as previously described[2] (Baek et al. *Methods and Protocols*).

References

1. Stoiber, M. De novo Identification of DNA Modifications Enabled by Genome-Guided Nanopore Signal Processing. . *bioRxiv* (2017).
2. Baek, A. et al. Mapping m(6)A Sites on HIV-1 RNA Using Oligonucleotide LC-MS/MS. *Methods Protoc* **7** (2024).
3. Ringeard, M., Marchand, V., Decroly, E., Motorin, Y. & Bennasser, Y. FTSJ3 is an RNA 2'-O-methyltransferase recruited by HIV to avoid innate immune sensing. *Nature* **565**, 500-504 (2019).
4. Tsai, K. et al. Acetylation of Cytidine Residues Boosts HIV-1 Gene Expression by Increasing Viral RNA Stability. *Cell Host Microbe* **28**, 306-312.e306 (2020).
5. Courtney, D.G. et al. Epitranscriptomic Addition of m(5)C to HIV-1 Transcripts Regulates Viral Gene Expression. *Cell Host Microbe* **26**, 217-227 e216 (2019).
6. Kennedy, E.M. et al. Posttranscriptional m(6)A Editing of HIV-1 mRNAs Enhances Viral Gene Expression. *Cell Host Microbe* **19**, 675-685 (2016).
