# Supplementary material for: Single-molecule epitranscriptomic analysis of full-length HIV-1 RNAs reveals functional roles of site-specific m6As
Source: Nat Microbiol. 2024 Apr 11;9(5):1340–55. doi: 10.1038/s41564-024-01638-5 (PMC11087264; doi:10.1038/s41564-024-01638-5)
Supplement: Supplementary file 2 — Reporting Summary [file 41564_2024_1638_MOESM2_ESM.pdf]

nature portfolio

Corresponding author(s): Sanggu Kim

Last updated by author(s): Jan 29, 2024

Reporting Summary

Nature Portfolio wishes to improve the reproducibility of the work that we publish. This form provides structure for consistency and transparency in reporting. For further information on Nature Portfolio policies, see our Editorial Policies and the Editorial Policy Checklist.

Statistics

For all statistical analyses, confirm that the following items are present in the figure legend, table legend, main text, or Methods section.

n/a	Confirmed	
☐	☒	The exact sample size (*n*) for each experimental group/condition, given as a discrete number and unit of measurement
☐	☒	A statement on whether measurements were taken from distinct samples or whether the same sample was measured repeatedly
☐	☒	The statistical test(s) used AND whether they are one- or two-sided<br>*Only common tests should be described solely by name; describe more complex techniques in the Methods section.*
☒	☐	A description of all covariates tested
☐	☒	A description of any assumptions or corrections, such as tests of normality and adjustment for multiple comparisons
☐	☒	A full description of the statistical parameters including central tendency (e.g. means) or other basic estimates (e.g. regression coefficient) AND variation (e.g. standard deviation) or associated estimates of uncertainty (e.g. confidence intervals)
☐	☒	For null hypothesis testing, the test statistic (e.g. *F*, *t*, *r*) with confidence intervals, effect sizes, degrees of freedom and *P* value noted<br>*Give P values as exact values whenever suitable.*
☒	☐	For Bayesian analysis, information on the choice of priors and Markov chain Monte Carlo settings
☒	☐	For hierarchical and complex designs, identification of the appropriate level for tests and full reporting of outcomes
☐	☒	Estimates of effect sizes (e.g. Cohen's *d*, Pearson's *r*), indicating how they were calculated

Our web collection on statistics for biologists contains articles on many of the points above.

Software and code

Policy information about availability of computer code

Data collection	QuantStudio 3D Digital PCR System was used for RT-PCR assays; Attune™ NxT flow cytometer (Thermo Fisher Scientific) for flow cytometry data collection; MinKNOW GUI (v3 or later; Nanopore technology) was used for sequencing data collection.
Data analysis	Multi-fast5 reads were base-called by guppy (version 3.2.8 or higher) and converted to single-read fast5s using the Oxford Nanopore Technologies API, ont_fast5 (v3.3.0). Fastqs were aligned with minimap2 (v2.24), and processed with SAMtools (v.1.6), NanoFilt (v2.7.1), and bedtools (v.2.25.0). The HIV-1 B subtype sequences for sequence conservation analysis were extracted from the HIV sequence database (https://www.hiv.lanl.gov/) and visualized using ggseqlogo R package (v.0.1). Tombo (v1.5.1), Eligos2 (v2.0.0), Nanocompore (v1.0.4), xPore (v2.1), Nanom6A (v2.0), and m6Anet (v-1.1.1) were used for RNA modification site calling. HIV-1 RNA splicing and poly(A) tail length analysis were conducted using minimap2 (v2.24), SAMtools (v.1.6), and Nanopolish (v.0.14.0). Blots were analyzed by ImageJ (v.1.53). Statistical analysis performed using GraphPad Prism9(v.9.5.0) or R package (v.4.0.2).

For manuscripts utilizing custom algorithms or software that are central to the research but not yet described in published literature, software must be made available to editors and reviewers. We strongly encourage code deposition in a community repository (e.g. GitHub). See the Nature Portfolio guidelines for submitting code & software for further information.

Data

Policy information about availability of data

All manuscripts must include a data availability statement. This statement should provide the following information, where applicable:

- Accession codes, unique identifiers, or web links for publicly available datasets
- A description of any restrictions on data availability
- For clinical datasets or third party data, please ensure that the statement adheres to our policy

All data supporting the findings of this study are available within the paper and its Supplementary Information. The Nanopore sequencing data used in this study were deposited into the European Nucleotide Archive (ENA) with an accession number PRJEB61077. The processed sequence data for each figure are available in Supplementary Information. The HIV-1 B subtype sequences corresponding to A8079, A8110, A8975 and A8989 of the NL4-3 strain (RNA) were extracted from the HIV sequence database (https://www.hiv.lanl.gov/).

Human research participants

Policy information about studies involving human research participants and Sex and Gender in Research.

Reporting on sex and gender	NA
Population characteristics	NA
Recruitment	NA
Ethics oversight	NA

Note that full information on the approval of the study protocol must also be provided in the manuscript.

Field-specific reporting

Please select the one below that is the best fit for your research. If you are not sure, read the appropriate sections before making your selection.

☒ Life sciences ☐ Behavioural & social sciences ☐ Ecological, evolutionary & environmental sciences

For a reference copy of the document with all sections, see nature.com/documents/nr-reporting-summary-flat.pdf

Life sciences study design

All studies must disclose on these points even when the disclosure is negative.

Sample size	We provide triplicated (or quadruplicated) experimental data of biologically independent samples. No statistical methods were used to pre-determine sample sizes, but the sample size of n=3 or n=4 routinely provide sufficient statistical power (when present) in our study utilizing accurate and highly reproducible assays and molecular biology experiments. These number of samples are commonly used in molecular biology publications to provide statistical conclusions, as well as to address the rigor and reproducibility.
Data exclusions	Low quality sequence reads were excluded by default QC threshold by MinKNOW (Nanopore technology). DRS cellular RNA runs occasionally showed poor read length distributions (Extended Data Fig.7a); when the fraction of > 2 Kb RNAs is less than 10% of the total reads, these samples were considered unsuitable and excluded from HIV-1 splicing analysis. For HIV-1 alternative splicing analysis, only the full-length reads were used (Fig. 4a).
Replication	Our data were highly reproducible in our repeated experiments using RNA samples that are independently prepared (n=3 or 4).
Randomization	Experimental groups were determined based on the experimental hypothesis (e.g. the impact of mutations or RNA isoforms) and all the experimental data and sequencing data that pass the data exclusion criteria were used without any additional selection.
Blinding	All the sequencing data that pass the data exclusion criteria were used without any additional selection. This study does not involve human studies. Data collection and analysis were not performed blind to the conditions of the experiments.

Reporting for specific materials, systems and methods

We require information from authors about some types of materials, experimental systems and methods used in many studies. Here, indicate whether each material, system or method listed is relevant to your study. If you are not sure if a list item applies to your research, read the appropriate section before selecting a response.

Materials & experimental systems

n/a	Involved in the study
☐	☒ Antibodies
☐	☒ Eukaryotic cell lines
☒	☐ Palaeontology and archaeology
☒	☐ Animals and other organisms
☒	☐ Clinical data
☒	☐ Dual use research of concern

Methods

n/a	Involved in the study
☒	☐ ChIP-seq
☐	☒ Flow cytometry
☒	☐ MRI-based neuroimaging

Antibodies

Antibodies used	Antibodies used for western blots are;<br>Anti-N6-methyladenosine (m6A) antibody (Abcam; Cat#ab208577) 1:1000 dilution<br>Anti-Gag(p24) (NIH AIDS Reagent; Cat#ARP-6458) 1:1000 dilution<br>Anti-Vif (NIH AIDS Reagent;cat# ARP-6459) 1:500 dilution<br>Anti-gp41(NIH AIDS reagent; cat# ARP-11391)1:500 dilution<br>Anti-Mouse HRP (Promega; cat#W4021)1:5000 dilution<br>Anti-GAPDH (Abcam; cat#ab8245) 1:1000 dilution
Validation	All antibodies were validated by respective vendors and obtained from the NIH AIDS Reagent program. These antibodies are commonly used and have been validated in multiple publications.<br>Anti-N6-methyladenosine (m6A) antibody (Abcam; Cat#ab208577) suitable for Northwestern, IP, Southern Blot. https://www.abcam.com/products/primary-antibodies/n6-methyladenosine-m6a-antibody-17-3-4-1-ab208577.html<br>Anti-Gag(p24) (NIH AIDS Reagent; Cat#ARP-6458),Anti-Vif (NIH AIDS Reagent;cat# ARP-6459) and Anti-gp41(NIH AIDS reagent; cat# ARP-11391) were obtained from NIH AIDS reagent Program and validated.<br>Anti-Mouse HRP (Promega; cat#W4021) suitable for WB. https://www.promega.com/products/protein-detection/primary-and-secondary-antibodies/anti_mouse-igg-h-and-l-hrp-conjugate/?catNum=W4021<br>Anti-GAPDH (Abcam; cat#ab8245) suitable for WB, ICC/IF. https://www.abcam.com/products/primary-antibodies/gapdh-antibody-6c5-loading-control-ab8245.html

Eukaryotic cell lines

Policy information about cell lines and Sex and Gender in Research

Cell line source(s)	HEK293T cells were purchased from ATCC; GHOST CXCR4+CCR5+ cells, Jurkat, and CEM-ss cell lines were from the NIH-supported HIV reagent program.
Authentication	HEK293T cells, GHOST CXCR4+CCR5+ cells, Jurkat, and CEM-ss cell lines are commonly used commercial cell lines distributed by ATCC and NIH-supported HIV reagent program, respectively; these cell lines were not further authenticated.
Mycoplasma contamination	HEK293T cells, GHOST CXCR4+CCR5+ cells, Jurkat, and CEM-ss cell lines used in this study were not tested for mycoplasma contamination.
Commonly misidentified lines (See ICLAC register)	No commonly misidentified cell lines were used in the study

Flow Cytometry

Plots

Confirm that:

☒ The axis labels state the marker and fluorochrome used (e.g. CD4-FITC).

☒ The axis scales are clearly visible. Include numbers along axes only for bottom left plot of group (a 'group' is an analysis of identical markers).

☒ All plots are contour plots with outliers or pseudocolor plots.

☒ A numerical value for number of cells or percentage (with statistics) is provided.

Methodology

Sample preparation	An equal amount of virus stock (pg) was used to infect GHOST cells in 6-well plates. After 48 h post-infection, the cells were washed with PBS three times and fixed with 3.7% formaldehyde for 10 minutes.
Instrument	The GFP expressions for all samples were acquired by the Attune NxT flow cytometer (Thermo fisher scientific)
Software	GFP expressions were analyzed by the FlowJo software (v 10.8.0) (BD biosciences)

Cell population abundance	The cell population abundance is shown in the relevant figures. Sorting was based on GFP.
Gating strategy	After dead cell removal based on the FSC/SSC gating, GFP+ cells were determined based on the gating two separate and distinct populations were visible in the relevant figures.

☒ Tick this box to confirm that a figure exemplifying the gating strategy is provided in the Supplementary Information.
