# Supplementary figures and images for: Single-molecule epitranscriptomic analysis of full-length HIV-1 RNAs reveals functional roles of site-specific m6As
Source: Nat Microbiol. 2024 Apr 11;9(5):1340–55. doi: 10.1038/s41564-024-01638-5 (PMC11087264; doi:10.1038/s41564-024-01638-5)

Figure 2a.

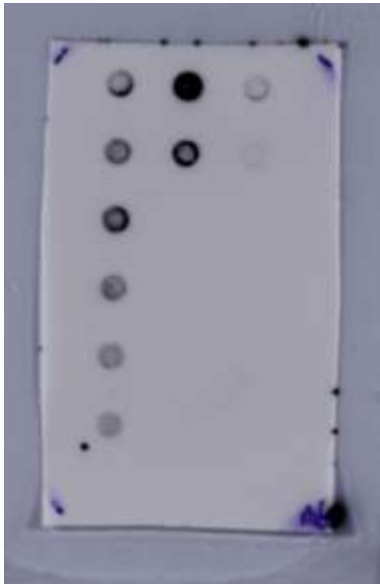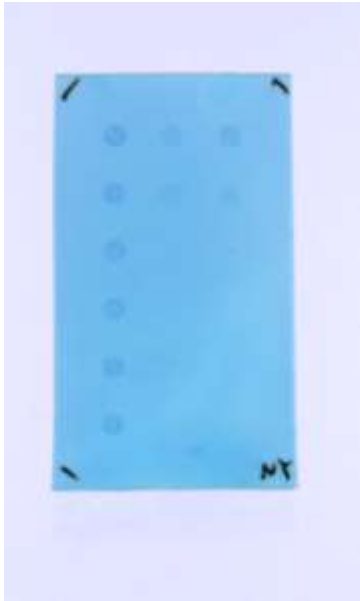

Figure 2c.

(ii)

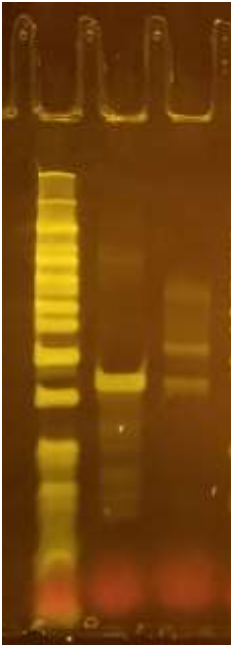

Supplement: Supplementary file 11 — Unprocessed gels/blots for figure. [file 41564_2024_1638_MOESM11_ESM.pdf]

Figure 3b.

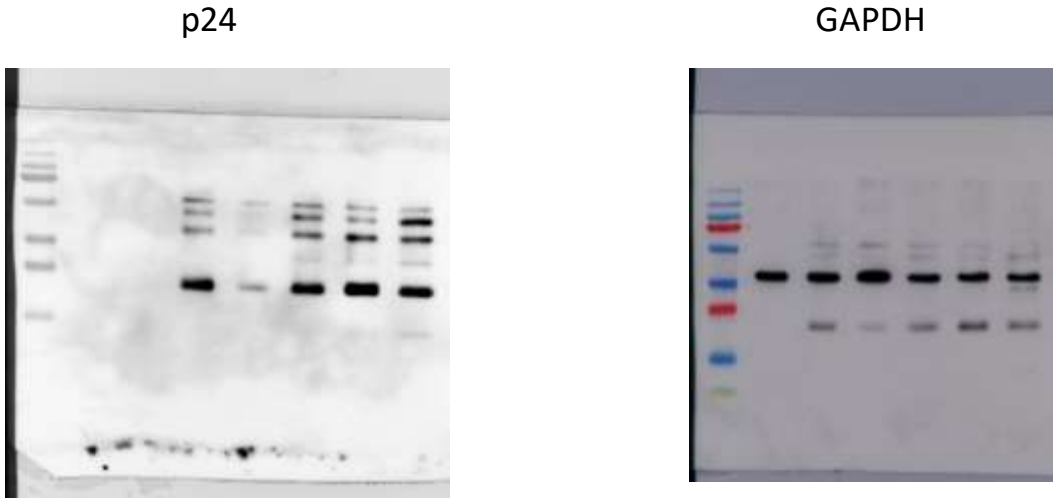

Figure 3c.

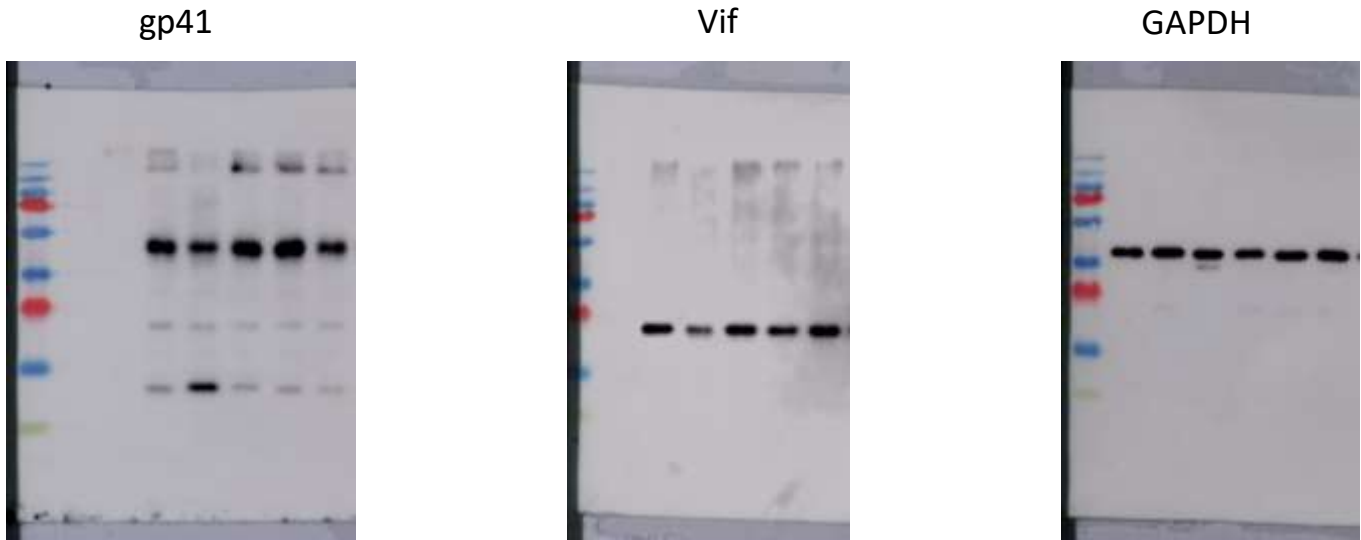

Supplement: Supplementary file 12 — Unprocessed blots for figure. [file 41564_2024_1638_MOESM12_ESM.pdf]

Figure 4c.

(i)

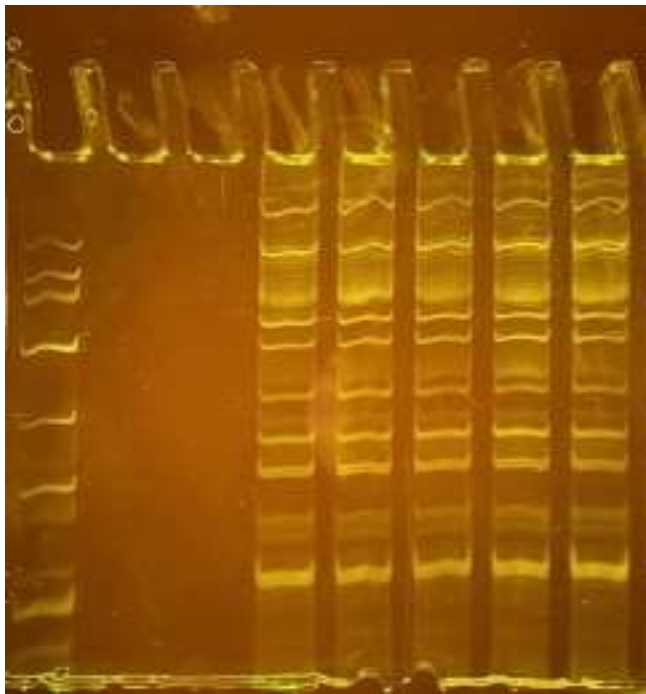

(ii)

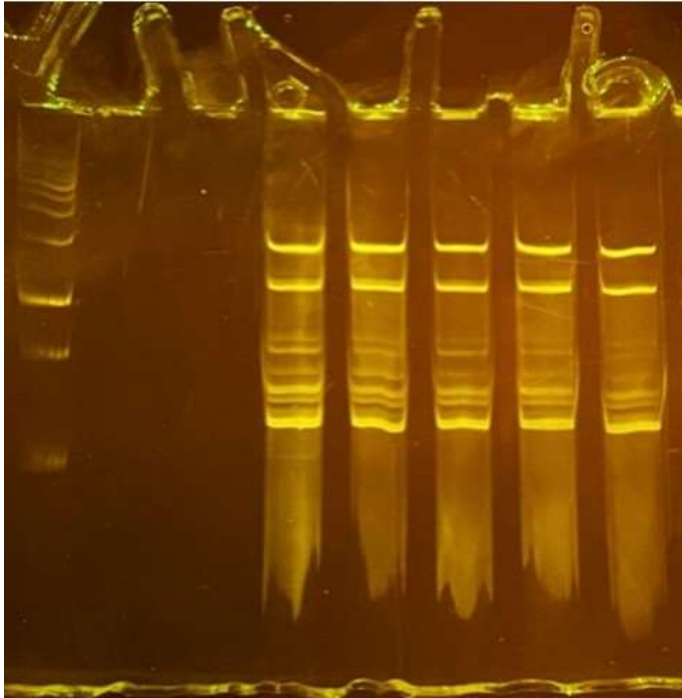

Supplement: Supplementary file 13 — Unprocessed gels for figure. [file 41564_2024_1638_MOESM13_ESM.pdf]
